# Newborn Screening for X-Linked Adrenoleukodystrophy: A Systematic Literature Review

**DOI:** 10.3390/ijns12040080

**Published:** 2026-09-30

**Authors:** María Isabel Cabrera-González, Yolanda De Diego-Otero, Raquel Yahyaoui

**Affiliations:** 1 Laboratorio de Metabolopatías y Cribado Neonatal, Hospital Regional Universitario de Málaga, 29011 Malaga, Spain; raquel.yahyaoui.sspa@juntadeandalucia.es; 2 Instituto de Investigación Biomédica de Málaga IBIMA Plataforma BIONAND, 29590 Malaga, Spain; 3 Departamento de Biología Molecular y Bioquímica, Universidad de Málaga, 29071 Malaga, Spain; 4 Departamento de Biomedicina y Odontología, Facultad de Ciencias Biomédicas y Deporte, Universidad Europea de Andalucía, 29010 Malaga, Spain

**Keywords:** systematic review, X-linked adrenoleukodystrophy, neonatal screening, newborn screening, peroxisomal disorders, C26:0-LPC and *ABCD1* gene

## Abstract

X-linked adrenoleukodystrophy (X-ALD) is the most common leukodystrophy and peroxisomal disorder. It was added to newborn screening (NBS) programs following its first implementation in New York in 2013. Since then, X-ALD screening has expanded rapidly, although important differences exist among programs. This systematic review aimed to evaluate the global implementation of NBS for X-ALD, including screening methodologies, screening performance, prevalence estimates, genetic findings, and clinical follow-up outcomes. A systematic search of PubMed and EMBASE identified 13 publications describing NBS programs conducted between 2013 and June 2026. All programs used C26:0-lysophosphatidylcholine (C26:0-LPC) as the primary biomarker, although substantial heterogeneity was observed in analytical methodologies, cut-off values, screening algorithms, and follow-up strategies. Positive predictive values ranged from 9.1% to 100%, while screening detection rates varied from 1:3118 to 1:51,081, reflecting differences in study design, screening population, sample size, and screening approaches. Variants of uncertain significance (VUS) accounted for 52.6% of individuals detected with classified *ABCD1* variant findings, representing a major challenge for genetic counseling and clinical management. Most identified newborns were asymptomatic at diagnosis, supporting the role of NBS in enabling early surveillance for adrenal insufficiency and cerebral disease before irreversible manifestations occur. In addition, differences between universal and sex-specific screening strategies highlight ongoing clinical and ethical debates regarding the scope of X-ALD NBS. Overall, current evidence supports the feasibility of NBS for X-ALD and its ability to enable presymptomatic ascertainment and early clinical surveillance; however, greater harmonization of screening protocols, improved variant interpretation, and long-term outcome studies are needed to optimize program performance and clinical benefit.

## 1. Introduction

X-linked adrenoleukodystrophy (X-ALD; OMIM: 300100) is the most common leukodystrophy and peroxisomal disorder, with an estimated prevalence of approximately 1:14,700 [1]. It is caused by pathogenic variants in the *ABCD1* gene, which encodes the adrenoleukodystrophy protein (ALDP), an ATP-binding cassette transmembrane protein. ALDP is involved in the transport of very long-chain saturated fatty acids (≥C22:0; VLCFAs) into the peroxisome for their degradation. Impairment of ALDP disrupts peroxisomal β-oxidation, leading to the accumulation of VLCFAs in plasma and tissues, affecting mainly the brain white matter, spinal cord, and adrenal cortex [1]. To date, more than 1290 unique *ABCD1* variants have been described. Traditionally, no clear genotype–phenotype correlation has been considered to exist, although emerging NBS data suggest that some variants may be associated with milder or later-onset phenotypes [2].

### 1.1. Clinical Manifestations

The clinical spectrum of X-ALD is highly heterogeneous. The three main phenotypes are adrenal insufficiency (AI), also called Addison’s disease (AD), adrenomyeloneuropathy (AMN), and cerebral adrenoleukodystrophy (cALD) [3].

AI predominantly affects males and is rare in females [4]. It is characterized by low or inappropriately normal cortisol levels in the presence of elevated adrenocorticotropic hormone (ACTH). Regular endocrine monitoring should begin during the first year of life and continue longitudinally, as untreated AI may lead to adrenal crisis, a potentially life-threatening medical emergency.

AMN represents the predominant neurological phenotype in adulthood and is characterized by a chronic, progressive myelopathy. In males, it typically presents between the second and fourth decades of life [5]. Clinical manifestations include spastic paraparesis, gait disturbances, peripheral neuropathy, and bladder dysfunction. Most male patients and a significant proportion of females develop AMN over time, although females generally present later and with milder symptoms [4,5,6,7]. AMN progression is generally slow, and management is supportive.

Cerebral adrenoleukodystrophy is the most severe phenotype, primarily affecting males. Approximately 35–40% of affected males develop cALD during childhood, between 5 and 12 years of age [8,9,10]. Brain magnetic resonance imaging (MRI) can detect inflammatory demyelination prior to clinical onset [11]. Early symptoms include cognitive, behavioral, and learning difficulties, followed by rapid neurological deterioration. Without timely treatment, such as hematopoietic stem cell transplantation (HSCT), the disease is typically fatal or results in severe disability within a few years of symptom onset [12].

### 1.2. Available Treatments

The management of X-ALD depends on the clinical phenotype and aims to prevent disease progression and manage symptoms. Patients with AI require lifelong glucocorticoid replacement therapy, with mineralocorticoid supplementation when indicated based on biochemical findings. Treatment of AMN is supportive and focuses on preserving functional ability and quality of life. Symptomatic management includes pharmacological treatment of neuropathic pain and spasticity, as well as multidisciplinary care involving rehabilitation and continence support. No curative treatment is currently available for this phenotype. Presymptomatic individuals identified through family screening require long-term surveillance. This includes regular endocrine evaluation for AI and periodic brain MRI, typically every six months during childhood and annually in adulthood, to detect early cerebral involvement [3]. International recommendations emphasize structured long-term surveillance of presymptomatic individuals, including regular adrenal assessment and serial brain MRI to enable early detection of treatable manifestations. Early identification of X-ALD is crucial because AI and cALD can benefit from timely surveillance and intervention.

The standard treatment for cALD is HSCT, which is most effective at an early stage of cerebral involvement, before the onset of neurological symptoms [3,4,5]. Outcomes are significantly poorer in advanced stages, particularly in patients with high Loes scores or established neurological impairment. In addition to HSCT, other emerging therapies, including lentiviral gene therapy, have shown promising results although their availability and regulatory status vary across regions [5,13]. Early detection is therefore critical to enable timely referral and intervention within the therapeutic window before the onset of irreversible neurological damage [5].

### 1.3. History of Newborn Screening for X-Linked Adrenoleukodystrophy

Newborn screening (NBS) enables the early detection of serious but treatable conditions. A substantial portion of disorders included in NBS panels are inborn errors of metabolism (IEM), also referred to as inherited metabolic disorders (IMD), which are individually rare but collectively represent a significant public health burden. Early detection of these conditions is crucial, as timely intervention can prevent irreversible disease progression [3].

X-ALD has been considered suitable for inclusion in NBS programs under contemporary interpretations of screening criteria, although its fit with the classical Wilson and Jungner principles is not complete. Early diagnosis enables timely initiation of adrenal hormone replacement therapy and surveillance for cerebral disease, allowing HSCT to be performed within a narrow therapeutic window, significantly improving outcomes [5,13].

The quantification of C26:0-lysophosphatidylcholine (C26:0-LPC) in dried blood spots (DBS) by liquid chromatography–tandem mass spectrometry (LC-MS/MS) represented a major advance in X-ALD NBS, while also enabling detection of other peroxisomal disorders (PD), establishing C26:0-LPC as a sensitive screening biomarker [14]. Further methodological refinements using negative-ion mode MS/MS with isocratic elution improved analytical specificity by reducing interference from other phosphocholine-containing lipids, including sphingomyelins and phosphatidylcholines, while maintaining high sensitivity for C26:0-LPC detection [15]. Alternatively, flow injection analysis–tandem mass spectrometry (FIA-MS/MS) has been widely used as a first-tier approach because it allows C26:0-LPC measurement to be multiplexed with the routine analysis of amino acids and acylcarnitines for inherited metabolic disorder screening.

**Figure 1:**
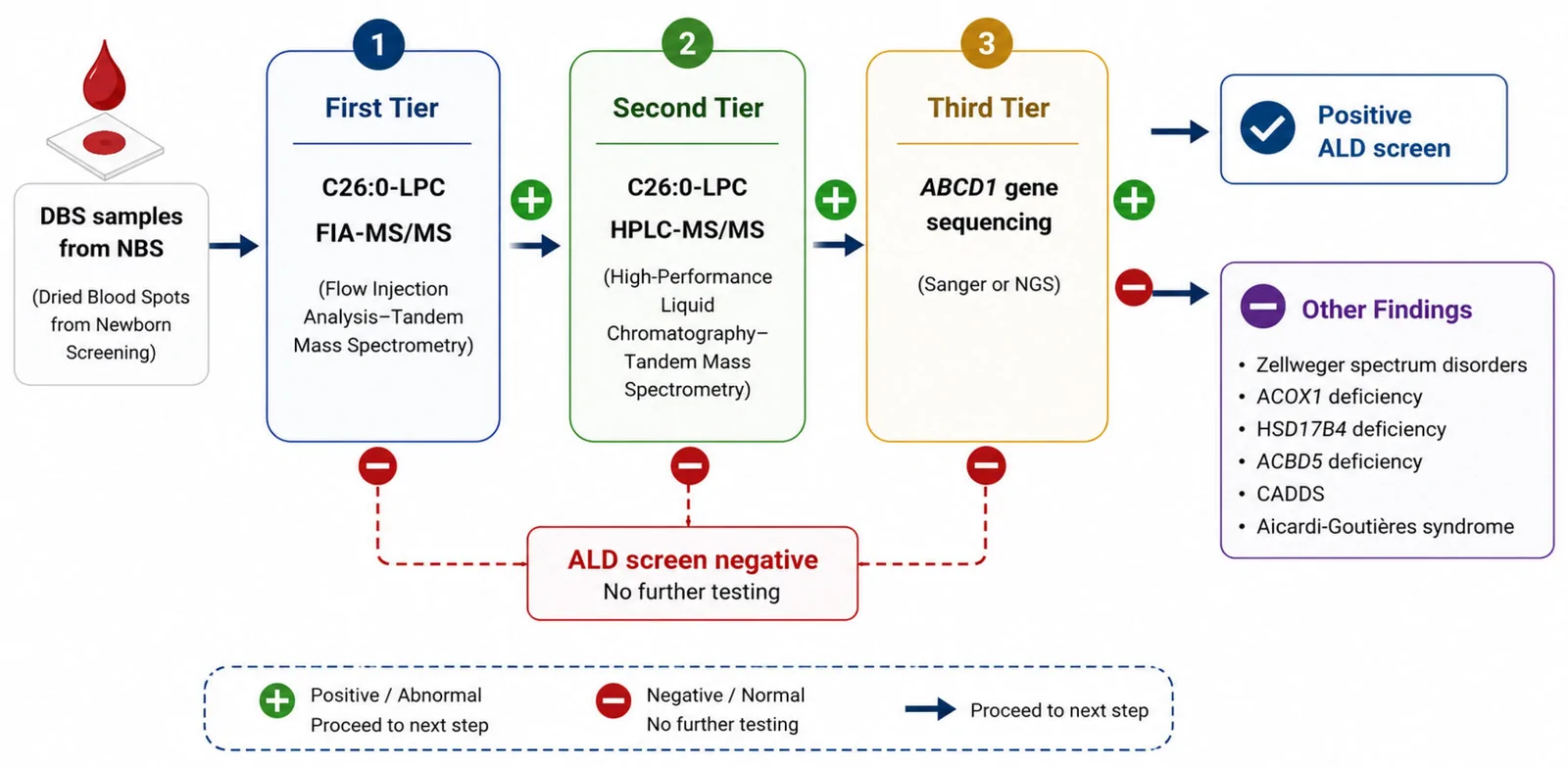
Multi-tier screening protocol for X-ALD using FIA-MS/MS, LC-MS/MS, and genetic confirmation. Adapted from Barendsen et al. [17].

New York became the first state to implement NBS for X-ALD in 2013 following the approval of Aiden’s Law. The screening program adopted a three-tier algorithm consisting of first-tier FIA-MS/MS measurement of C26:0-LPC, second-tier LC-MS/MS confirmation, and third-tier *ABCD1* sequencing for newborns with persistently abnormal biochemical results [16]. Most NBS programs have subsequently adopted comparable multi-tier screening approaches that combine biochemical and genetic testing (Figure 1) [16,17,18].

Following its inclusion in the Recommended Uniform Screening Panel (RUSP) in 2016, NBS for X-ALD has expanded rapidly, enabling the identification of affected individuals prior to symptom onset and facilitating early diagnosis and timely intervention, particularly HSCT within the therapeutic window. In addition to affected males, screening programs may also identify females with *ABCD1* variants and newborns with other PD. However, variability persists across programs, including differences in biochemical cut-off values and the implementation of sex-specific versus universal screening strategies.

In 2021, the Netherlands initiated a pilot screening program restricted to male newborns [17,18]; X-ALD was subsequently incorporated into the Dutch routine newborn screening program in 2023. Growing interest in the implementation of NBS for X-ALD has also been documented across several European countries, including Spain, Slovenia, Ireland, and other regions [19]. More recently, pilot screening studies have been reported in Italy [20], Japan [21], and Southern China [22].

Important differences exist among national screening strategies. The Dutch program differs substantially from those implemented in most other countries, as it combines a sex-specific approach with a four-tier screening algorithm. First, C26:0-LPC is measured in DBS by FIA-MS/MS. In the second tier, the sex of the newborn is determined using the X-counter assay, allowing only male infants to proceed through the screening pathway. The third tier consists of a repeat measurement of C26:0-LPC using a more sensitive and specific analytical method and a lower cut-off value. Finally, sequencing of the *ABCD1* gene is performed to identify pathogenic variants associated with X-ALD. This strategy was designed to identify males at risk of developing cALD while minimizing the detection of conditions for which preventive treatment is currently unavailable. Similarly, the Japanese X-ALD NBS programs were conducted in specific regions, including Aichi Prefecture, Gifu Prefecture, and the Clinical and Research Association for Rare, Intractable Diseases (CReARID), a multi-regional network encompassing several participating areas across the country, and was performed exclusively in male newborns [21]. However, unlike the Dutch strategy, male eligibility was defined at enrolment, and no dedicated sex-determination step was incorporated into the screening algorithm.

The growing international adoption of X-ALD NBS has led to considerable heterogeneity in screening policies and methodologies, highlighting the need for a comprehensive synthesis of the available evidence. This systematic review therefore aims to critically evaluate the global implementation and outcomes of NBS for X-ALD. Literature published from 2013 onwards was included, corresponding to the introduction of X-ALD screening in New York, the first NBS program to incorporate this disorder. Specifically, this review aims to characterize screened populations, screening strategies, biomarkers, cut-off values, performance, population coverage, prevalence estimates, genetic findings, and available clinical follow-up data in X-ALD NBS programs. The present study applies a systematic review methodology, extends the available evidence through June 2026, and provides a structured comparison of published X-ALD NBS programs, with particular emphasis on PPV and SPR, sex-specific detection rates, *ABCD1* variant classification, secondary findings, and available clinical follow-up.

## 2. Methodology

### 2.1. Literature Search

This systematic review was conducted and reported in accordance with the Preferred Reporting Items for Systematic Reviews and Meta-Analyses (PRISMA) 2020 guidelines. A comprehensive literature search was performed in PubMed and EMBASE on 18 June 2026 to identify studies on NBS for X-ALD worldwide.

**Figure 2:**
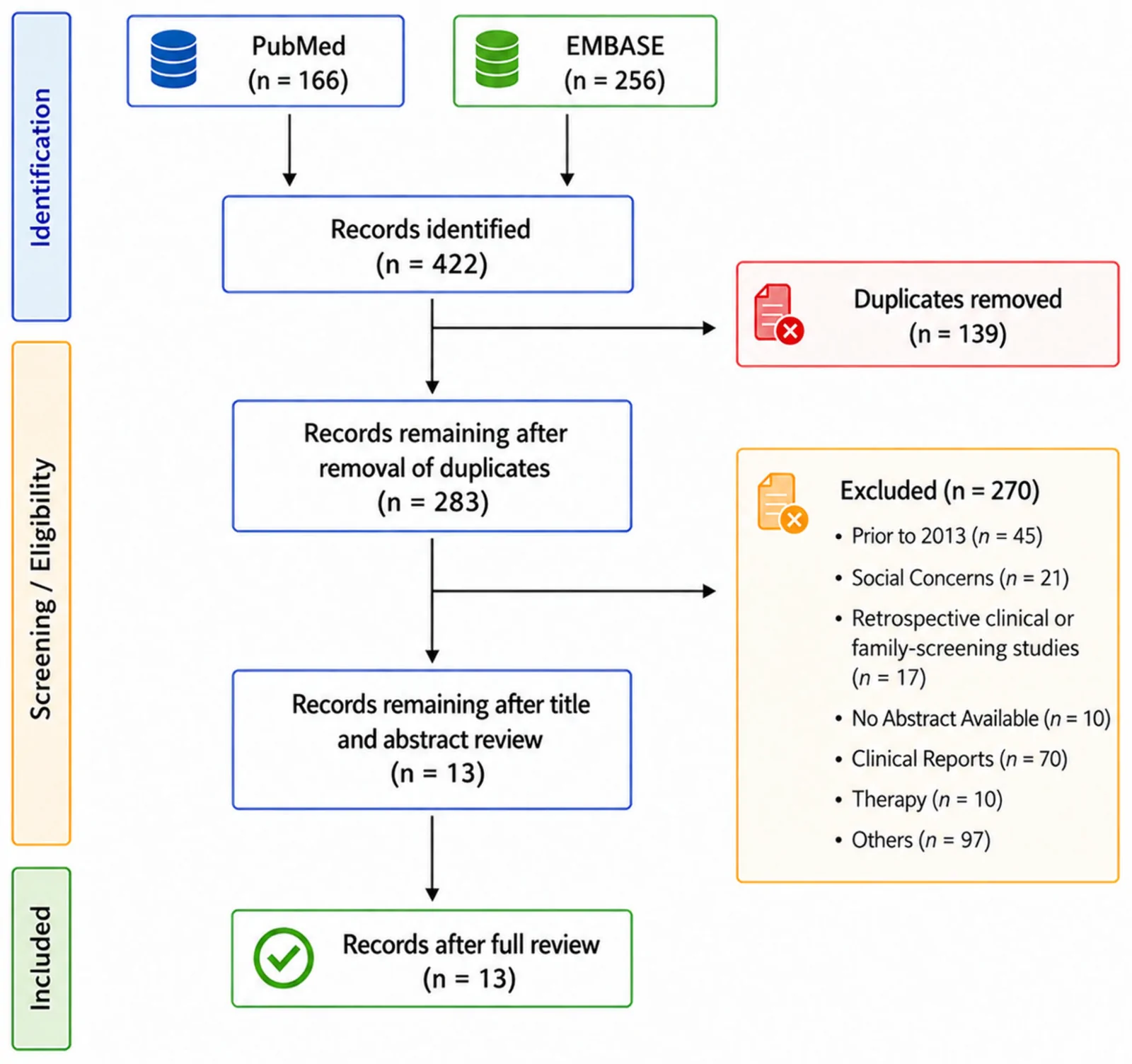
PRISMA 2020 flow diagram of the study selection process.

The search strategy included the terms (“adrenoleukodystrophy*” OR “ALD” OR “X-ALD”) AND (“newborn” OR “neonatal”) AND (“screening” OR “screen*”). The search was restricted to the title and abstract fields. Only articles published in English were included. Titles and abstracts were screened independently to assess eligibility according to predefined inclusion and exclusion criteria. The study identification and selection process is summarized in the PRISMA flow diagram (Figure 2).

Two reviewers (R.Y. and Y.D.O.) independently screened titles and abstracts and subsequently assessed the full texts of potentially eligible studies. Disagreements were resolved by consensus.

### 2.2. Eligibility Criteria

Studies were included if they met the following criteria: (i) reported original data from NBS for X-ALD; (ii) described pilot, regional, national, or population-based NBS programs conducted in previously undiagnosed newborns; (iii) reported screening outcomes, including the number of newborns screened, screen-positive cases, confirmed X-ALD diagnoses and prevalence estimates; and (iv) were published between 2013 and June 2026, corresponding to the period following the implementation of the first X-ALD newborn screening program in New York State.

Duplicate publications, review articles, systematic reviews, meta-analyses, guidelines, consensus statements, editorials, commentaries, letters, case reports, conference abstracts, and publications without an available abstract were excluded. Publications were also excluded if they did not report original newborn screening data, focused exclusively on clinically diagnosed patients or family screening, or addressed only ethical, social, economic, or laboratory methodological aspects without reporting screening results. Retrospective analyses of newborn screening programs were eligible if they met the inclusion criteria.

When multiple publications described the same NBS program or overlapping cohorts, data were not considered as independent observations. The most comprehensive or most recent report was used for each outcome, while earlier publications were retained when they provided methodological or screening-performance information not reported in subsequent updates.

### 2.3. Data Collection

Data extraction was performed independently by two reviewers using a standardized data extraction form. Data were extracted and organized into three predefined tables.

For Table 1, data related to the screening protocol were extracted, including country and region, first-, second-, and third-tier screening methodologies, biomarkers analyzed, corresponding cut-off values, the use of genetic testing within the screening algorithm, genetic testing methods (when applicable), and other relevant methodological characteristics reported by the authors. When methodological details were not provided in the included study, data were retrieved from the original methodological publication referenced by that study. Screening tiers were defined as sequential analytical steps within the NBS algorithm that provided additional analytical information beyond simple repeat measurement. Repeat measurement using the same analytical method, whether performed on the initial specimen or on a second specimen, was considered repeat testing within the same tier rather than an additional screening tier. Genetic testing was considered a screening tier only when performed before classification as screen-positive; genetic testing performed after screen-positive classification was considered part of diagnostic follow-up. Borderline results, requests for a second specimen, and other relevant repeat-testing procedures were recorded as additional methodological characteristics of each screening program.

For Table 2, study-level characteristics were extracted, including country and region, year of publication, study population size, total, male, and female newborns screened, study duration, screen-positive newborns, X-ALD-positive screening outcomes, screen-positive rate (SPR), and positive predictive value (PPV). SPR was defined as the proportion of screened newborns referred for confirmatory evaluation after a positive screening result (screen-positive newborns/total newborns screened). PPV was defined as the proportion of screen-positive newborns subsequently confirmed with X-ALD or carrying an *ABCD1* variant classified as pathogenic, likely pathogenic, or a variant of uncertain significance (VUS), relative to the total number of screen-positive newborns identified by the screening algorithm. In the Minnesota study, one newborn in whom no *ABCD1* variant was detected showed reduced ALDP function; for the purposes of the present review, this case was therefore considered an X-ALD-related positive outcome and included as a true-positive case in the PPV calculation.

To ensure consistency across studies, screening outcomes were harmonized whenever sufficient data were available. Newborns in whom an *ABCD1* variant was identified in the context of a positive biochemical screen were considered X-ALD-positive screening outcomes regardless of variant classification (pathogenic, likely pathogenic, or VUS), whereas newborns diagnosed with other peroxisomal disorders were not considered X-ALD-positive outcomes. The inclusion of VUS in this definition reflects the screening context rather than molecular confirmation of disease: these variants were identified following an abnormal biochemical screening result and represent clinically relevant screening outcomes that may require additional biochemical evaluation, genetic counseling, family studies, and longitudinal surveillance. Accordingly, VUS were included as positive screening outcomes for PPV calculations but were distinguished throughout the analysis from molecularly confirmed pathogenic or likely pathogenic *ABCD1* variants. PPV was recalculated according to these standardized criteria whenever sufficient data were available. Incomplete confirmatory follow-up among screen-positive newborns may have affected PPV estimates and, in some programs, resulted in underestimation of the actual PPV.

For Table 3, clinical and genetic outcomes were collected, including overall and sex-specific X-ALD screening detection rates; classification of *ABCD1* variants as pathogenic, likely pathogenic, or VUS; other peroxisomal disorders detected through NBS; and clinical outcomes reported during follow-up. Screening detection rates were calculated as the proportion of screened newborns with an X-ALD-positive screening outcome according to the definition above and should therefore not be interpreted as estimates of molecularly confirmed X-ALD birth prevalence.

### 2.4. Quality Assessment

The methodological quality of the included studies was independently assessed by two reviewers using the Joanna Briggs Institute (JBI) Critical Appraisal Checklist for Studies Reporting Prevalence Data. The checklist evaluates nine domains addressing sample representativeness, participant recruitment, sample size, description of the study population and setting, coverage of the identified sample, validity and consistency of the methods used for identification of the condition, statistical analysis, and response rate. Disagreements between reviewers were resolved by consensus.

## 3. Results

### 3.1. Study Selection

A total of 422 records were identified through PubMed (*n* = 166) and EMBASE (*n* = 256). After removal of 139 duplicates, 283 records underwent title and abstract screening. Of these, 270 were excluded according to the predefined eligibility criteria, leaving 13 articles for full review. All 13 met the eligibility criteria and were included in the final analysis (Figure 2) [1,18,20,21,22,23,24,25,26,27,28,29,30]. These studies reported original X-ALD NBS data from the United States, the Netherlands, Italy, Japan, China, and Taiwan.

### 3.2. Geographical Coverage of X-ALD NBS Programs

The geographical coverage of X-ALD NBS varied considerably across studies, ranging from nationwide implementation to regional pilot initiatives. In the United States and the Netherlands screening was implemented through statewide and nationwide programs, respectively. In contrast, several studies were based on regional initiatives. The Italian study was conducted as a regional pilot program in the Lombardy Region, while the Chinese study was carried out in Guangzhou, Guangdong Province (Southern China). In Japan, screening was restricted to selected regional initiatives, including Aichi Prefecture and the network CReARID. In Taiwan, screening was conducted through the National Taiwan University Hospital Newborn Screening Center, which screens approximately 30–35% of newborns nationwide [25].

### 3.3. Screening Methodologies

Considerable variability was observed in the analytical approaches used across newborn screening programs. For first-tier screening, FIA-MS/MS was the most commonly used platform and was used in programs from New York [1], Italy [20], the Netherlands [17,18], Pennsylvania [24], Taiwan [25], Nebraska [26], California [27], and Georgia [29]. In contrast, LC-MS/MS was used as the primary screening method in programs from Japan [21], China [22], Illinois [23], North Carolina [28], and Minnesota [30] (Table 1). In addition, second-tier testing included LC-MS/MS, which was the most frequently applied method, either as a dedicated second-tier assay or as part of the biochemical follow-up pathway after an initial positive result. Several screening programs also incorporated molecular genetic testing as a third-tier analysis. Notably, the Netherlands designates this molecular analysis as a fourth-tier test because its screening algorithm includes an additional intermediate tier based on X-counter analysis.

### 3.4. Biomarkers and Metabolites

All included NBS programs used C26:0-LPC as the primary biomarker for X-ALD. Five programs additionally incorporated C24:0-LPC into the screening algorithm, including those conducted in Italy [20], Japan [21], North Carolina [28], Georgia [29], and Illinois [23], where C24:0-LPC was used for the evaluation of borderline samples.

Two programs expanded the biomarker panel beyond C26:0-LPC and C24:0-LPC. The Italian program included C20:0-, C22:0-, C24:0-, and C26:0-LPC together with selected VLCFA-related acylcarnitines, whereas the Georgia program incorporated C20:0-, C22:0-, C24:0-, and C26:0-LPC [29]. In addition, the Italian and Georgia programs incorporated Collaborative Laboratory Integrated Reports (CLIR) as part of the post-analytical evaluation process [20,29,31].

### 3.5. Cut-Off Values

Cut-off values for C26:0-LPC varied across programs. Borderline ranges were defined in three studies, namely those conducted in Illinois [23], North Carolina [28], and Minnesota [30].

Three programs using first-tier LC-MS/MS defined borderline ranges, with thresholds ranging from 0.08 µM to 0.29 µM. Among studies that did not define a borderline category, positive cut-off values varied according to the analytical platform employed. In general, FIA-MS/MS-based protocols used higher C26:0-LPC cut-off values than LC-MS/MS-based protocols, which typically applied lower thresholds (Table 1).

**Table 1:** Newborn screening strategies, biomarkers, analytical methods, and cut-off values for X-linked adrenoleukodystrophy.

	1st Tier			2nd Tier			3rd Tier Genetic Testing		
Population	Method	Biomarkers	Cut-Offs (µM)	Method	Biomarkers	Cut-Offs (µM)		Method	Additional Information
Italy [20]	FIA-MS/MS	C20:0-, C22:0-, C24:0-, C26:0-LPC, and C20:0-, C22:0-, C24:0-, C26:0- acylcarnitine.	C26:0-LPC≥ 0.5 CLIR analysis	LC-MS/MS	C26:0-LPC C26:0- carnitine	≥0.1	No	NA	Two-tier screening algorithm; second tier was performed on the same well as the first tier [32]. The first-tier C26:0-LPC cut-off was reduced from 0.65 to 0.50 µM [20].
Japan [21]	LC-MS/MS	C24:0-LPC and C26:0-LPC	NA	-	-	-	No	NA	Regional program. Male-only NBS conducted by CReARID, Aichi Prefecture, and Gifu Prefecture. A second DBS was requested for repeat testing. The screening algorithm was described previously [33].
China [22]	LC-MS/MS	C26:0-LPC	≥0.17	-	-	-	No	NA	Pilot study. Positive samples were retested in duplicate without requesting a second DBS. NanoWES with Sanger confirmation was performed during diagnostic follow-up.
Illinois [23]	LC-MS/MS	C26:0-LPC	1st repeat ≥ 0.16 Borderline 0.18–0.28 Positive: ≥0.28	-	-	-	No	NA	Statewide NBS program. Borderline results required a second DBS; specimens with C24:0-LPC <0.02 µM were considered invalid. Genetic testing was performed during diagnostic follow-up.
Pennsylvania [24]	FIA-MS/MS	C26:0-LPC	≥0.36	LC-MS/MS	C26:0-LPC	≥0.15	Yes	*ABCD1* gene testing (NGS)	Statewide NBS program. A second DBS was requested for repeat second-tier testing before reflex *ABCD1* sequencing. A PBD gene panel was performed in *ABCD1*-negative cases during diagnostic follow-up.
Taiwan [25]	FIA-MS/MS	C26:0-LPC	≥0.3	LC-MS/MS	C26:0-LPC	≥0.4	Yes	*ABCD1* gene testing (NGS)	Regional program. All three tiers were performed using the same DBS. Reflex WES was performed for persistent C26:0-LPC elevation with negative *ABCD1* sequencing. The program started in November 2016, and the third tier was introduced in August 2019.
Nebraska [26]	FIA-MS/MS	C26:0-LPC	≥0.36	LC-MS/MS	C26:0-LPC	≥0.15	Yes	*ABCD1* gene testing (long-range PCR+ NGS)	Statewide NBS program. Second-tier testing was performed on the same initial DBS; a second DBS was requested for repeat second-tier testing before reflex *ABCD1* sequencing.
Netherlands [18]	FIA-MS/MS	C26:0-LPC	≥0.32	2nd tier X-counter 3rd tier LC-MS/MS	C26:0-LPC	≥0.15	Yes	4th-tier *ABCD1* gene testing (Sanger sequencing)	Pilot sex-specific NBS program with a 4-tier algorithm: an X-counter was incorporated as the 2nd tier to select newborns with one X chromosome before LC-MS/MS analysis.
California [27]	FIA-MS/MS	C26:0-LPC	≥0.42	LC-MS/MS	C26:0-LPC	≥0.22	Yes	*ABCD1* gene testing (Sanger sequencing)	Statewide NBS program. The second-tier cut-off was increased from 0.15 to 0.22 µM in December 2017. Confirmatory follow-up includes VLCFAs and plasmalogen analysis, deletion/duplication testing, and additional molecular testing (e.g., PEX genes).
North Carolina [28]	LC-MS/MS	C24:0-LPC C26:0-LPC	Borderline:C24:0-LPC ≤ 0.175 C26:0-LPC ≥ 0.08–0.15 Positive: C26:0-LPC ≥ 0.15 or C26:0-LPC 0.08–0.15 C24:0-LPC ≥ 0.175	-	-	-	No	NA	Statewide pilot NBS program. Samples above the initial cut-off were retested in duplicate using the same LC-MS/MS assay. A repeat specimen was requested for borderline results. *ABCD1* sequencing was performed after classification as screen-positive.
Georgia [29]	FIA-MS/MS	C20, C22, C24, C26-LPC	CLIR analysis	LC-MS/MS	C26:0-LPC	>0.30	No	NA	Pilot study. CLIR cut-off: informative score > 1st percentile of confirmed cases. Confirmatory follow-up: plasma VLCFAs + *ABCD1* sequencing or other PBD genes.
Minnesota [30]	LC-MS/MS	C26:0-LPC	Borderline: 0.16–0.29 Positive: ≥0.3	-	-	-	No	NA	Statewide screening program. Samples with a C26:0-LPC value ≥0.30 μmol/L were considered screen positive and the patient was referred for further testing and diagnostic evaluation. Samples with a C26:0-LPC value between 0.16 and 0.29 μmol/L were considered screen borderline, and a repeat heel-stick specimen was requested. If this repeat sample was again ≥0.16 μmol/L, the infant was considered screen positive. Confirmatory follow-up: serum VLCFAs analysis + *ABCD1* mutation analysis; not part of the screening algorithm.
New York [1]	FIA-MS/MS	C26:0-LPC	NA	LC-MS/MS	C26:0-LPC	NA	Yes	*ABCD1* sanger sequencing	Statewide screening program. If *ABCD1* is negative, additional genetic and biochemical testing for other PD is performed as follow—up.

CLIR, Collaborative Laboratory Integrated Reports; DBS, dried blood spot; WES, whole-exome sequencing; NA, not available; PD, peroxisomal disorders; PBD, peroxisomal biogenesis disorders; FIA-MS/MS, flow injection analysis–tandem mass spectrometry; LC-MS/MS, liquid chromatography–tandem mass spectrometry; VLCFAs, very-long-chain fatty acids; C26:0-LPC, C26:0-lysophosphatidylcholine; PCR, polymerase chain reaction.

Additional cut-off values for C24:0-LPC were reported in programs that incorporated this biomarker into their screening algorithms. However, the thresholds varied across studies according to the analytical platform and screening strategy used (Table 1).

### 3.6. Population Coverage and Screening Performance

Relevant differences were observed among X-ALD NBS programs regarding population size and screening performance. California reported the largest cohort, screening more than 1.8 million newborns over four years [27]. Large-scale population-based programs were also reported in New York [1], Pennsylvania [24], and Japan [21], each screening several hundred thousand newborns. Although smaller in size, the Nebraska statewide program screened more than 80,000 newborns over 3 years and 4 months [26].

The SPR and the PPVs varied considerably across programs, reflecting differences in screening algorithms, cut-off values, and the implementation of second- and third-tier testing. The highest PPVs were reported in the Netherlands [18] and Minnesota [30] (100%), followed by Nebraska [26] (92.9%), Pennsylvania [24] (86.3%), New York [1] (81.1%), and Italy [20] (78.6%). Intermediate PPVs were observed in Taiwan [25] (71%), California [27] (66.5%), North Carolina [28] (50%), and Illinois [23] (44.1%). The lowest PPVs were reported in Japan [21] (19.8%) and Georgia [29] (9.1%). In China [22], the PPV was recalculated as 43.8% according to the methodology applied in this review; however, this estimate should be interpreted with caution because of substantial loss to follow-up among screen-positive newborns (Table 2).

**Table 2:** Characteristics and outcomes of X-ALD newborn screening programs.

Population/Region	Year	Study Size			Screen- Positive (*n*)	SPR (%)	PPV (%)	Study Length
		Total	Males	Females				
Italy (Lombardy) [20]	2025	138,116	70,854	67,255	14	0.0101	78.6	3 y, 9 m
Japan (Aichi, Gifu, CReARID) [21]	2025	138,038	138,038	NS	86	0.0623	19.8	>1 y *
China (Guangzhou, Guangdong) [22]	2024	43,653	23,270	20,383	32	0.0733	43.8 **	1 y, 6 m
Illinois [23]	2022	276,000	NA	NA	34	0.0123	44.1 ****	1 y, 11 m
Pennsylvania [24]	2022	542,554	278,330	264,224	51	0.0094	86.3	4 y, 2 m
Taiwan [25]	2022	320,528	NA	NA	31	0.0097	71	1 y, 6 m
Nebraska [26]	2022	82,920	42,332	40,588	14	0.0169	92.9	3 y, 4 m
Netherlands [18]	2022	71,208	NA	NS	4	0.0056	100	1 y
California [27]	2021	1,854,631	945,344	900,917	355	0.0191	66.5	4 y
North Carolina [28]	2020	52,301	26,443	25,026	12	0.0229	50 ***	5 m
Georgia [29]	2020	51,081	NA	NA	11	0.0215	9.1	6 m
Minnesota [30]	2019	67,835	33,976	32,005	14	0.0206	100	1 y
New York [1]	2016	630,000	NA	NA	53	0.0084	81.1	2 y, 8 m

Year, year of publication; SPR, screen-positive rate; PPV, positive predictive value; y, years; m, months; NA, not available; NS, not screened. Differences between the total number of screened newborns and the sum of male and female newborns reflect cases for which sex information was unavailable. This applied to 7 newborns in Italy, 8370 in California, >1% of screened newborns in North Carolina, and 1854 newborns in Minnesota. * Duration of individual screening programs was not uniformly reported; all participating programs had conducted NBS for >1 year. ** For China, PPV was recalculated according to the harmonized definition used in this review (14/32; 43.8%). However, this estimate should be interpreted with caution because of substantial loss to follow-up among screen-positive newborns. The original study reported a PPV of 80% based only on the five infants who completed confirmatory testing (4/5). *** The original study reported a PPV of 67%, which included X-ALD and other genetic disorders; for consistency across studies, PPV was recalculated according to the harmonized definition of X-ALD-positive screening outcomes used in this review. **** The original study considered two additional cases as X-ALD-positive that did not meet the harmonized definition used in this review: one had not undergone genetic testing and one had pending extended genetic analysis.

### 3.7. Variants of Uncertain Significance (VUS)

VUS were frequently identified across X-ALD NBS programs. Overall, 205 of 390 individuals with available *ABCD1* variant classification carried a VUS (52.6%). The nationwide Japanese survey identified 16 *ABCD1* VUS, whereas only one newborn was diagnosed with X-ALD [21]. In the Nebraska program, the high frequency of VUS was specifically highlighted as a major difficulty in genetic counseling and clinical follow-up [26]. A similarly high proportion of VUS among screen-positive newborns was observed in Pennsylvania (41.2%; 21/51) [24] and California (41.4%; 147/355) [27].

In contrast, no VUS were identified among the 11 newborns diagnosed with X-ALD in the Italian pilot study; five *ABCD1* variants were classified as pathogenic and six as likely pathogenic [20]. In Minnesota [30], only one of 12 genetically characterized cases (8.3%) carried a VUS. One case had no detectable *ABCD1* variant and genetic results were unavailable for another case, whereas all remaining variants were classified as pathogenic or likely pathogenic. Similarly, no VUS were reported in the Taiwanese program, where all *ABCD1* variants were classified as pathogenic or likely pathogenic. Notably, null variants accounted for 8 of the 22 confirmed X-ALD cases [25].

Most studies implemented follow-up protocols including plasma VLCFAs measurement and genetic counseling when a VUS was identified (Table 3).

Accordingly, newborns with an *ABCD1* VUS identified in the context of a positive biochemical screen were distinguished from molecularly confirmed X-ALD cases but were not considered conventional false-positive screening results, as they may require additional diagnostic evaluation and longitudinal surveillance.

### 3.8. Detection of Other Peroxisomal Disorders Through X-ALD NBS

X-ALD NBS also enabled the identification of other disorders associated with elevated very-long-chain fatty acid biomarkers (Table 3). Zellweger spectrum disorders (ZSD) were the most frequently reported secondary diagnoses, with variants identified in *PEX1*, *PEX5*, *PEX10*, and *PEX26*. Other peroxisomal disorders included D-bifunctional protein (DBP) deficiency (*HSD17B4*), acyl-CoA oxidase 1 (ACOX1) deficiency, and unspecified peroxisomal biogenesis disorders (PBDs). In addition, the North Carolina program identified one case of Aicardi–Goutières syndrome associated with a *TREX1* variant. In contrast, the Netherlands implemented a sex-specific screening strategy incorporating an X-counter to select newborns with one X chromosome before subsequent biochemical testing, thereby limiting the detection of non-X-ALD conditions.

**Table 3:** X-ALD screening detection rates by sex, *ABCD1* variant classifications, other peroxisomal disorders detected, and clinical manifestations reported during follow-up.

Population	X-ALD Screening Detection Rate			*ABCD1* Variants, *n*			Clinical Manifestations, *n* (%)		Other Peroxisomal Disorders
	Males	Females	Total	VUS	P	LP	cALD	AD	Type, *n*
Italy [20]	1:11,809	1:13,451	1:12,556	0	5	6	0	4 (36.4%)	ZSD, *n* = 2 (PEX10, *n* = 1; PEX 26, *n* = 1); ACOX1 deficiency (*n* = 1)
Japan [21]	1:8120	NS	1:8120	16	1	0	NA	NA	NA
China [22]	1:3324	1:2911	1:3118	5	0	9	0	0	ZSD, (*PEX1*, *n* = 1); DBP deficiency (*HSD17B4*, *n* = 1)
Illinois [23]	NA	NA	1:18,400	8	7	0	NA	NA	Other PD, *n* = 3 (*PEX1*-related PBD, *n* = 1; DBP deficiency (*HSD17B4*, *n* = 1); unspecified PD (*n* = 1).
Pennsylvania [24]	1:13,000	1:11,000	1:12,330	21	21	2	0	4	PBD not specified, *n* = 4
Taiwan [25]	1:13,825	1:15,463	1:14,569	0	10	12	0	2	ZSD (*PEX1*, *n* = 2); DBP deficiency (*HSD17B4*, *n* = 1)
Nebraska [26]	1:10,583	1:4510	1:6378	4	9	0	0	0	NA
Netherlands [18]	1:9000	NS	1:17,802	0	1	3	NA	NA	NS
California [27]	1:9090 *	1:6825 *	1:7859 *	147	59	25	0 **	14 (20%)	ZSD, *n* = 23
North Carolina [28] ***	1:8814	1:8342	1:8717	3	2	1	0	2	ZSD (*PEX1*, *n* = 1)
Georgia [29]	NA	ND	1:51,081	0	1	0	NA	NA	ZSD, *n* = 2 (PEX1, *n* = 1; PEX5, *n* = 1)
Minnesota [30] ****	1:3878	1:6586	1:4845	1	3	8	0	0	ND
New York [1]	NA	NA	1:14,700	NA	NA	NA	NA	NA	NA

*ABCD1* variants, *n*: number of individuals by variant classification; Clinical manifestations, *n*: patients with clinical manifestations during follow-up; NA, not available; ND, no cases detected during the study period, prevalence could not be estimated; NS, not screened; VUS, variant of uncertain significance; LP, likely pathogenic; P, pathogenic. Screening detection rates (SDRs) were calculated using the population eligible for screening under each program design and are therefore not directly comparable across universal and sex-specific screening strategies. In Japan, X-ALD screening was restricted to male newborns; consequently, the male and total SDRs are identical and refer exclusively to the male population screened. In the Netherlands, all newborns underwent first-tier C26:0-LPC screening, but only newborns identified as having one X chromosome by the second-tier X-counter proceeded through the X-ALD screening algorithm. Accordingly, the male SDR (~1:9,000) refers to screened boys, whereas the overall SDR (1:17,802) represents the yield relative to the entire first-tier screened population. In universal screening programs, the overall SDR includes both male and female newborns. * For California, sex-specific and overall screening detection rates were recalculated according to the harmonized definition used in this review, including *ABCD1* variants identified during confirmatory follow-up. In addition to variants detected by initial Sanger sequencing, confirmatory testing identified four *ABCD1* deletions (one male and three females) and one mosaic variant (male). These additional variants were included in the calculation of screening detection rates but could not be assigned to the pathogenic or likely pathogenic categories because their specific classification was not reported. ** Five patients developed abnormal brain MRI findings, and one of them also showed neurological symptoms; however, no confirmed cases of cALD requiring HSCT were reported. *** Sex-specific detection rates for North Carolina were calculated from the sex-specific denominators reported in the original study (26,443 males and 25,026 females). The published demographic counts include 880 newborns with other/unknown sex and show a minor numerical discrepancy with the reported total study population (52,301); therefore, the overall detection rate was calculated using the reported total population, whereas sex-specific rates used the corresponding published sex-specific denominators. **** For Minnesota, clinical outcomes correspond to the follow-up period reported in the study included in this review. A subsequent publication in 2025 reported longer-term follow-up and additional clinical outcomes from the same screening program; this later report was not included to avoid duplication of an overlapping cohort.

### 3.9. Clinical Findings and Follow-Up Outcomes

Most newborns identified through screening were asymptomatic at the time of diagnosis. In Minnesota [30], all newborns diagnosed with X-ALD showed no evidence of adrenal insufficiency (AI) or cerebral involvement at the time of reporting, with the oldest patient being approximately 24 months of age. Similarly, the only infant diagnosed with X-ALD in Georgia [29] was asymptomatic at diagnosis, with no reported AI or cerebral disease; however, the patient was subsequently lost to follow-up. Comparable findings were also reported in China [22] (Table 3).

Among the included studies, the California program [27] provided the most comprehensive long-term follow-up data. Clinical outcomes were available for 71 of the 95 males diagnosed with X-ALD through NBS. Of these, 14 (20%) developed adrenal involvement, including five (7%) who required glucocorticoid replacement therapy. Brain MRI abnormalities were identified in five patients (7%), although only one child developed neurological symptoms during follow-up. No confirmed cases of childhood cALD requiring HSCT were reported during the study period.

## 4. Discussion

This systematic review identified 13 publications describing NBS programs for X-ALD, showing the rapid expansion of its screening since its first implementation in New York in 2013 [1,16]. However, substantial heterogeneity persists regarding analytical methodologies, cut-off values, screening algorithms, genetic confirmation strategies, and follow-up protocols. These findings highlight the absence of international standardization and complicate direct comparisons between programs.

One of the most relevant findings of this review was the variability in analytical approaches. Although all screening programs used C26:0-LPC as the primary biomarker, first-tier methodologies differed considerably. Most programs employed FIA-MS/MS as the initial screening method [1,18,20,24,25,26,27,29], whereas LC-MS/MS was used as the first-tier approach in Japan, China, Illinois, North Carolina, and Minnesota [21,22,23,28,30]. This preference for FIA-MS/MS likely reflects its lower operational cost and compatibility with existing NBS workflows, despite its lower analytical specificity compared with chromatographic methods [14,15]. LC-MS/MS was widely used either as a first-tier method or as a dedicated second-tier assay, depending on the screening algorithm [1,18,20,21,22,23,24,25,26,27,28,29,30].

Despite the use of similar multistep algorithms, the definition of a positive screening result was not consistent across studies. In some programs incorporating third-tier *ABCD1* gene sequencing, screen-positive cases corresponded to infants referred after second-tier biochemical testing, whereas others defined positivity only after completion of third-tier genetic testing. These methodological differences complicate direct comparisons of detection rates and PPVs across screening programs.

Differences in the integration of molecular testing were also associated with substantial variation in PPV. Taiwan and Nebraska combined biochemical screening with *ABCD1* gene sequencing before referral, reducing false-positive results and improving screening efficiency. In Taiwan [25], sequencing results were integrated with second-tier biochemical findings before performing confirmatory diagnosis, whereas Nebraska required persistent biochemical abnormalities on repeat testing followed by *ABCD1* gene sequencing. In contrast, California reported a lower PPV (66.5%) together with a high proportion of VUS. After increasing the second-tier C26:0-LPC cut-off, both the proportion of VUS and the observed prevalence decreased, highlighting the influence of screening thresholds on diagnostic yield. Overall, these findings suggest that combining molecular testing with optimized cut-off values may improve screening specificity, although potentially at the expense of reduced detection of individuals with an X-ALD variant and lower C26:0-LPC concentrations. The 100% PPV reported in Minnesota may partly reflect first-tier LC-MS/MS and repeat DBS testing for borderline results; however, this estimate was based on only 14 screen-positive newborns and should be interpreted cautiously. The particularly low PPV reported in Georgia should also be interpreted cautiously, as it was derived from a small pilot study with only 11 screen-positive newborns.

Beyond methodological differences, important variation exists in screening policies. Most programs adopted universal screening approaches including both male and female newborns [20,22,23,24,25,26,27,28,29,30]. However, sex-specific screening strategies were implemented in the Netherlands and in the regional Japanese programs, where screening was restricted to male newborns only [18,21]. The Dutch program incorporated the X-counter assay to identify male newborns before biochemical confirmation, whereas the Japanese programs [21] preselected male newborns using sex information recorded at birth. This distinction reflects an ongoing ethical and clinical debate. Current NBS biomarkers cannot predict the future clinical phenotype of screen-positive newborns. Therefore, both universal and male-only screening programs inevitably identify males who may remain asymptomatic for decades or develop only adult-onset AMN, for which no disease-modifying treatment is currently available. However, these individuals cannot currently be distinguished at birth from those who will develop adrenal insufficiency or cerebral disease, for which early surveillance and timely intervention may substantially improve outcomes. This represents an important consideration when X-ALD screening is evaluated in the context of classical screening criteria. Universal screening facilitates the identification of females with *ABCD1* variants and at-risk relatives through cascade testing and may also increase detection of other PDs [18]. Although females were historically referred to as carriers, current evidence indicates that a substantial proportion develop AMN-like manifestations in adulthood. Further studies comparing the long-term clinical and psychosocial consequences of both approaches are needed to inform future policy decisions. Methodological differences between programs should also be considered when interpreting prevalence estimates, as variations in cut-off values and referral algorithms may influence case ascertainment and potentially lead to underdetection of individuals with lower C26:0-LPC concentrations, particularly heterozygous females.

An additional source of heterogeneity was the role of molecular testing. While several programs incorporated *ABCD1* gene sequencing as a third-tier component of the NBS algorithm, others—including Illinois, Minnesota, and Georgia—performed molecular analysis only during confirmatory diagnostic evaluation following a positive biochemical screen. In addition, the Dutch program incorporated an X-chromosome counting assay as a second-tier step to select newborns with one X chromosome before subsequent biochemical testing and fourth-tier *ABCD1* sequencing. Consequently, the definition of a screen-positive result was not consistent across studies, complicating direct comparisons of referral rates and PPVs across screening programs.

Another important difference between screening programs concerns the detection of other peroxisomal disorders. Most algorithms enabled the identification of disorders beyond X-ALD, particularly ZSD (*PEX1*, *PEX5*, *PEX10* and *PEX26*), D-bifunctional protein deficiency (*HSD17B4*), and *ACOX1* deficiency, through additional biochemical and/or genetic testing. In contrast, the Dutch algorithm was intentionally designed to identify only X-ALD; therefore, newborns with positive biochemical results but no *ABCD1* variant were not investigated further [18]. However, the included studies did not systematically evaluate the clinical or psychosocial consequences of identifying these secondary conditions, limiting assessment of the broader benefits and potential harms of their detection through X-ALD NBS.

The increasing implementation of X-ALD NBS has also led to the identification of a growing number of *ABCD1* variants, including VUS [34]. More than 1290 unique *ABCD1* variants have been reported to date [2]. Overall, across the studies included in this review, 205 of the 390 individuals with classified *ABCD1* variant findings carried a VUS (52.6%), representing one of the major challenges encountered across screening programs [21,22,23,24,26,27,28,34]. Some programs used the ABCD1 Variant Registry as a reference to determine whether identified *ABCD1* variants were likely to be pathogenic or VUS [22,26,28]. In addition, differences in the definition of screen-positive cases and in whether VUS were considered positive screening outcomes significantly affected prevalence and PPV estimates. Diagnostic criteria were not fully standardized across programs, with some studies incorporating clinical, biochemical and familial information in addition to molecular findings when establishing a final diagnosis, which may have influenced estimates of confirmed X-ALD cases and PPV. Notably, for Japan [21], 16 VUS and one ALD diagnosis were included as positive screening outcomes according to the harmonized criteria of this review, whereas the authors originally reported a PPV of only 1.2%. For descriptive purposes, VUS were included in the summary of reported *ABCD1* variants because they represent clinically relevant molecular findings that may be identified in newborns with biochemical abnormalities consistent with X-ALD, although their diagnostic interpretation remains uncertain and often requires additional biochemical, functional, familial, and longitudinal data.

The high frequency of VUS identified through NBS further highlights the importance of long-term follow-up studies [2]. As screened cohorts mature, longitudinal follow-up may help determine whether these variants are ultimately associated with the development of clinical manifestations or remain clinically silent over time, thereby reducing diagnostic uncertainty and improving genetic counseling. International collaborative registries and long-term natural history studies will be particularly important to address this challenge [35]. This diagnostic uncertainty has recently been addressed by the Grey Zone Project, which included contributions from the Italian and Dutch NBS programs and proposes a risk-based framework integrating *ABCD1* variant interpretation with biochemical and longitudinal clinical data [36].

A major unmet need in X-ALD NBS is the identification of reliable biomarkers capable of predicting clinical phenotype from birth. Despite early diagnosis, it remains impossible to accurately determine which individuals will develop AI, cALD, AMN, or remain asymptomatic for prolonged periods [4]. The discovery of prognostic biomarkers could enable risk-stratified follow-up protocols, allowing more intensive surveillance for individuals at higher risk while reducing the burden of monitoring in those with a lower likelihood of developing severe manifestations. Future research should therefore focus not only on improving screening performance but also on identifying biochemical, genetic, imaging, or multi-omic markers associated with disease progression and phenotype prediction. In addition, biomarkers capable of predicting not only disease phenotype but also age at disease onset would further improve surveillance strategies and clinical decision-making. Importantly, molecular pathogenicity does not necessarily predict clinical severity, and some pathogenic ABCD1 variants may be associated with mild or very late-onset phenotypes. Genotype, particularly when integrated with neonatal C26:0-LPC concentrations, may ultimately contribute to prognostic stratification, although long-term validation is required.

Despite these challenges, the available evidence supports the feasibility of X-ALD NBS and its ability to identify individuals presymptomatically, providing an opportunity for structured clinical surveillance. Most newborns identified through screening were asymptomatic at diagnosis, demonstrating the ability of NBS to detect affected individuals before the onset of irreversible disease manifestations. Some programs identified presymptomatic AI, allowing early initiation of corticosteroid replacement before an adrenal crisis [20,21,24,25,27,28].

This review has several limitations. Only studies published in English were included, potentially excluding relevant data from other regions. In addition, considerable heterogeneity in study design, analytical methodologies, and reporting practices precluded quantitative synthesis or meta-analysis. Although all programs screened large populations, ranging from 43,653 newborns [22] to more than 1.8 million [27], differences in the frequency and distribution of *ABCD1* variants among populations may contribute to variability in detection rates. Furthermore, methodological differences in first-tier screening strategies, including analytical platforms, cut-off values, and the definition of borderline results, may influence screening sensitivity and potentially affect the identification of some affected newborns, particularly those with biochemical values close to established thresholds. Moreover, definitions of screen-positive cases and confirmatory diagnostic criteria differed between studies, limiting direct comparisons of PPVs and screening performance across programs.

Some X-ALD NBS pilot studies have been recently implemented or remain in pilot phases, and their results have only been reported in conference abstracts, conference proceedings or other non–peer-reviewed sources without detailed methodological or outcome data [19,37,38]. In addition, long-term clinical outcome data remain scarce because most screened cohorts are still relatively young, limiting assessment of the full impact of NBS on morbidity and mortality. Importantly, published study periods ranged from approximately five months to just over four years, while individual clinical follow-up was variably reported and generally short relative to the lifelong natural history of X-ALD. Consequently, current studies cannot establish lifetime false-negative rates or the ultimate clinical significance of many screen-detected individuals. Moreover, despite more than a decade of implementation, X-ALD NBS remains marked by substantial geographical inequities. Screening is not universally available and is often restricted to specific countries, regions, or populations, with some programs screening only male newborns. Consequently, current evidence may not be fully representative of the global X-ALD population, underscoring the need for broader implementation and longer follow-up.

## 5. Conclusions

NBS for X-ALD has expanded over the last decade and current evidence supports its feasibility, presymptomatic ascertainment and the opportunity for early clinical surveillance. Measurement of C26:0-LPC in DBS enables the identification of affected individuals before the onset of irreversible neurological or endocrine manifestations, allowing timely surveillance and intervention for AI and cerebral disease.

However, important challenges remain. Considerable variability persists among programs regarding analytical methodologies, screening algorithms, population coverage, genetic confirmation strategies, and follow-up practices, limiting direct comparisons across studies. The high proportion of VUS and the currently limited ability to predict phenotype and age at onset from genotype or neonatal biochemical findings continue to complicate clinical interpretation, prognostic assessment, and genetic counseling. Furthermore, substantial disparities persist in the implementation of X-ALD NBS across countries, regions, and target populations, and long-term outcome data remain limited.

Future efforts should focus on harmonizing screening protocols and reporting standards, improving variant classification frameworks, expanding equitable access to screening, and establishing long-term international follow-up cohorts. Such initiatives will be essential to fully define the clinical impact of NBS for X-ALD and to maximize its benefits for affected individuals and their families.

## Data Availability

No new data were created or generated in this study. All data analyzed are available in the published articles included in this systematic review.

## Abbreviations

ABCD1	ATP-binding Cassette Subfamily D Member 1
ACTH	Adrenocorticotropic Hormone
AD	Addison’s Disease
AGS	Aicardi–Goutières syndrome
AI	Adrenal Insufficiency
ALDP	Adrenoleukodystrophy Protein
AMN	Adrenomyeloneuropathy
ZSD	Zellweger Spectrum Disorder
X-ALD	X-linked Adrenoleukodystrophy
cALD	Cerebral adrenoleukodystrophy
CLIR	Collaborative Laboratory Integrated Reports
CReARID	Clinical and Research Association for Rare, Intractable Diseases
C24:0-LPC	C24:0-lysophosphatidylcholine
C26:0-LPC	C26:0-lysophosphatidylcholine
DBS	Dried Blood Spot
FIA-MS/MS	Flow Injection Analysis Tandem Mass Spectrometry
HPLC-MS/MS	High-Performance Liquid Chromatography-Tandem Mass Spectrometry
HSCT	Hematopoietic Stem Cell Transplantation
IEM	Inborn Errors of Metabolism
IMD	Inherited Metabolic Disorders
LPC	Lysophosphatidylcholine
MRI	Magnetic Resonance Imaging
MS/MS	Tandem Mass Spectrometry
NBS	Newborn Screening
PBD	Peroxisomal Biogenesis Disorders
PD	Peroxisomal Disorders
PPV	Positive Predictive Value
PRISMA	Preferred Reporting Items for Systematic Reviews and Meta-Analyses
RUSP	Recommended Uniform Screening Panel
VLCFAs	Very Long-Chain Fatty Acids
VUS	Variant of Uncertain Significance
WES	Whole Exome Sequencing
